# A novel application of tau PET in the diagnosis of sporadic inclusion body myositis: A case report

**DOI:** 10.1097/MD.0000000000021524

**Published:** 2020-07-31

**Authors:** Yutong Zhang, Ke Li, Chuanqiang Pu, Haodan Dang, Jiajin Liu, Qiang Shi

**Affiliations:** a Department of Neurology, The First Medical Centre, Chinese PLA General Hospital; b Department of Neurology, Aerospace Central Hospital; c Department of Neurology, The Second Medical Centre, Chinese PLA General Hospital; d Department of Nuclear Medicine, The First Medical Centre, Chinese PLA General Hospital; e Department of National Clinical Research Center of Geriatrics Disease, Chinese PLA General Hospital, Beijing, China.

**Keywords:** sporadic inclusion body myositis, Tau PET/MRI, THK5317

## Abstract

**Rationale:**

Sporadic inclusion body myositis (sIBM) is a chronic progressive idiopathic inflammatory myopathy, with characteristic rimmed vacuoles and sarcoplasmic abnormal tau protein deposits. THK5317, an ^18^F-labelled positron emission tomography (PET) marker, targets tau protein deposits, which are expressed in the brain of patients with Alzheimer's disease (AD). It is assumed that THK5317 PET/MRI may also depict tau protein in the skeletal muscles of patients with sIBM. Here we introduced a novel application of tau PET in diagnosis of sIBM in a rare case.

**Patient concerns:**

We presented a 46-year-old woman who suffered from progressive lower limb weakness for one and a half year.

**Diagnoses:**

Needle electromyography showed myogenic damage. Characteristic myopathological changes of sIBM were discovered, and abnormal tau protein deposits were identified by tau immunostaining. Genetic testing ruled out the GNE myopathy, a hereditary distal myopathy with rimmed vacuoles. The patient was finally diagnosed as sIBM.

**Interventions:**

We performed [^18^F] THK5317 PET/MRI on the patient.

**Outcomes:**

There were significantly increased tau uptake levels in the quadriceps muscles of sIBM patient. The uptake levels of tau in the quadriceps were significantly higher than that in the posterior group of thigh muscles, which was consistent with the distribution characteristics of involved muscle groups.

**Lessons:**

[^18^F] THK5317 PET can reveal muscular tau deposition in vivo, which provides a new and noninvasive diagnostic method for sIBM and offers the opportunity to monitor the progression of tau pathology along with muscle impairment.

## 1 Introduction

Sporadic inclusion body myositis (sIBM) is described as an uncommon, progressive, inflammatory myopathy, mostly involving patients older than 50.^[1]^ Forearm flexors and quadriceps are the most commonly affected muscles. Further research investigated that a variety of abnormal protein deposits similar to those in the brain of patients with Alzheimer's disease (AD) have been discovered in sIBM muscle fibers, such as phosphorylated tau protein. This suggests that sIBM and AD have similar pathogenesis and pathological characteristics.^[2]^ In vivo study on patients with AD using [^18^F] THK5317 has shown considerable cortical uptake and temporal lobe retention, in comparison to healthy control individual. Based on the similarities of pathologic features shared by sIBM and AD, we tentatively display tau protein deposits in vivo by using [^18^F] THK5317 PET/MRI in a female patient with typical pathological features of sIBM. This is the first reported sIBM case who was performed by [^18^F] THK5317 PET/MRI and muscle biopsy.

This study passed the ethical review by the Medical Ethics Committee of Chinese PLA General Hospital.

## 2 Case presentation

A 46-year-old woman was presented to our hospital with progressive lower limb weakness for one and a half year with no family history of neuromuscular diseases. Her previous medical history was normal. Neurological examination showed normal speech and mental status, and the cranial nerve examination was normal. Manual muscle strength testing was entirely normal in the upper extremities, and lower in bilateral proximal (grade 3) and distal (grade 4) of pelvic limbs. Neck flexor and extensor strength were normal. Deep tendon reflexes were normal, without Hoffman signs or clonus, and plantar responses were flexor. The sensory examination was normal. No truncal or appendicular ataxia was noted. Serum creatine kinase (CK) level was 689.6 U/L (<200 U/L in normal level), and lactic dehydrogenase (LDH) level was 243.7 U/L (<250 U/L in normal level).

**Figure 1:**
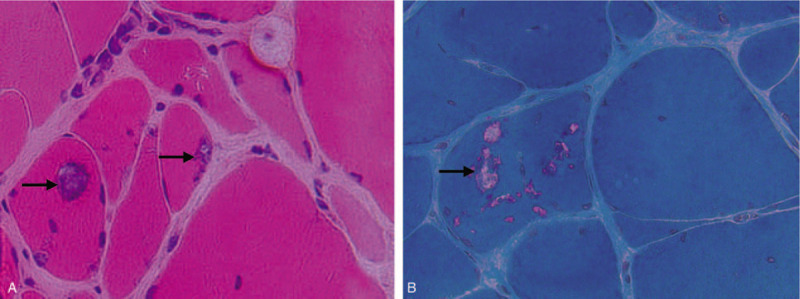
Rimmed vacuoles and inclusion bodies revealed by muscle biopsy. (A) Mononuclear cell invasion and rimmed vacuoles (arrow) were seen (H&E, 400×). (B) Inclusion bodies of some rimmed vacuoles (arrow) in fibers were stained as red (Gomori trichrome staining, 400×).

**Figure 2:**
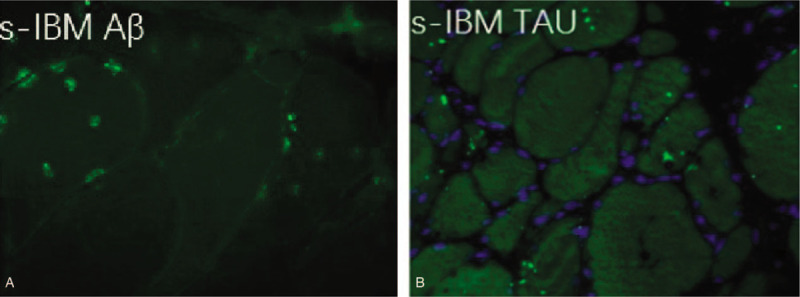
Positive immunofluorescent staining of amyloid protein (A) and tau protein (B).

**Figure 3:**
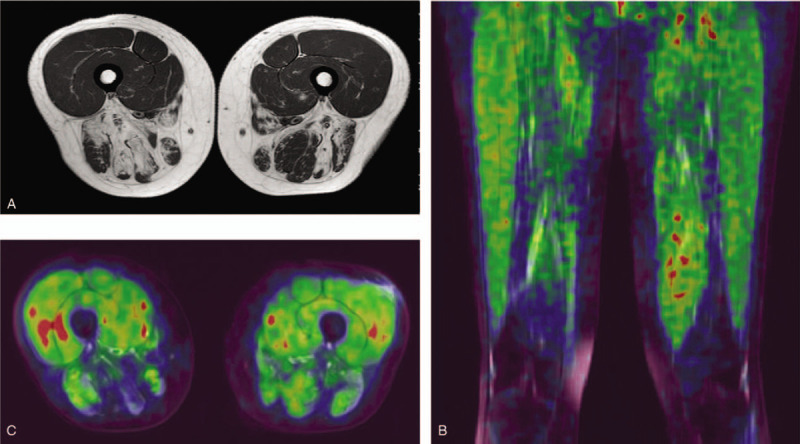
MRI & PET images. (A) MRI image showed atrophy and partial fatty transformation of bilateral lower limb muscles. (B) Tau PET images showed elevated radioactive uptake in the bilateral quadriceps. (C) PET axial image revealed the SUV levels of quadriceps = 0.73/0.67 (right/left).

The patient's nerve conduction studies were normal. Her needle electromyography demonstrated fibrillation potentials in quadriceps and anterior tibialis muscle, with small motor unit potentials and rapid recruitment in these and upper and lower extremities muscles, including first interosseous muscle, quadriceps and anterior tibialis muscle. Muscle biopsy of the left quadriceps muscle revealed some necrotic fibers, rimmed vacuoles in muscle fibers with basophilic deposits (Fig. 1A, B), and mononuclear inflammatory cells invading non-necrotic muscle fibers. These findings were consistent with the diagnosis of sIBM. Furthermore, immunofluorescent staining for Aβ and tau protein were displayed dense deposition (Fig. 2A, B). Magnetic resonance imaging (MRI) showed a partial fatty transformation of the bilateral lower limb muscles (Fig. 3A). Next-generation sequencing was performed, and the result ruled out the glucosamine (UDP-N-acetyl)-2-epimerase/N-acetylmannosamine kinase (GNE) myopathy, a hereditary distal myopathy with rimmed vacuoles.

Subsequent [^18^F] THK5317 PET/MRI was performed on the patient after she signed the informed consent. Forty minutes after intravenous injection of [^18^F] THK5317, whole body distribution of radioactivity was measured with a Siemens PET/MRI scanner (MAGNETOM Biograph mMR). PET image reconstruction based on MRI was performed to analyze the difference of radioactive uptake in muscles. Reading of [^18^F] THK5317 PET/MRI image was performed by at least two nuclear medicine physicians, looking for focally enhanced uptake within skeletal muscles. The case presented with increased [^18^F] THK5317-SUV levels in the biopsy site of the left quadriceps with corresponding tau positive immunostaining. Tau uptakes of bilateral quadriceps were increased to SUV level of 0.73/0.67 (right/left) (Fig. 3B, C). Meanwhile there was no obvious tau retention in the posterior group of thigh muscle with SUV level below than 0.5. Tau uptake in the quadriceps was significantly higher than that in the posterior group of thigh muscles, which was consistent with the distribution characteristics of involved muscle groups.

## 3 Discussion

Sporadic inclusion body myositis is a distinct progressive inflammatory skeletal muscular disease. The quadriceps weakness is the most common clinical symptom in sIBM patients. Characteristic muscle biopsy findings in inclusion body myositis include auto aggressive endomysial inflammation, rimmed vacuoles and sarcoplasmic abnormal protein deposits.^[3]^ The muscle pathological manifestations of sIBM are similar to Alzheimer's disease, that is, abnormal deposition of multiple proteins, such as tau protein and amyloid protein. Due to advances in molecular imaging, it has been reported that tau PET could improve diagnostic efficiency for Alzheimer's disease.^[4]^ However, there is no report on the application of tau PET in inclusion body myositis so far.

In our study, we introduced a novel application of tau PET in diagnosis of sporadic inclusion body myositis in a case which is diagnosed with pathological definite sIBM. To our knowledge, this is the first report on using of the tau PET/MRI within sIBM patient, which presented a significantly increased uptake of tau protein within involved muscles. This suggests that [^18^F] THK5317-PET can depict muscular tau deposits in vivo. Although muscle biopsy can reveal abnormal deposition of tau protein in sIBM, but it cannot provide the condition of tau deposition within all skeletal muscles. Our case further confirms that the muscles of patients with sIBM can show tau-tracer binding in vivo. The application of tau PET has allowed identification of tau deposition in vivo and can help to improve for the ability and efficiency of sIBM diagnosis.

Maetzler et al previously reported that ^11^C PIB-PET amyloid imaging can depict abnormal deposits of amyloid protein in sIBM patient, but there was no correlation between the radioactive uptake level and the clinical severity of the involved muscles.^[5]^ Recently a new study by Lilleker et al^[6]^ suggested that another amyloid PET imaging, which detects amyloid using the radioactive tracer 18F-florbetapir, is helpful for the differential diagnosis of inclusion body myositis and polymyositis. In our study, we discovered that THK5317 SUV levels within quadriceps muscles were increased than those of other involved muscles, which consistent with the clinical severity of involved muscles. It suggested that tau PET imaging could visually analyze the deposition of tau protein in the muscles of patients with inclusion body myositis and reflected the severity of involvement muscles. Tau PET offers the opportunity to monitor the progression of tau pathology along with muscle impairment. These findings suggest that [^18^F] THK5317 could be an excellent imaging agent for tau protein in sIBM patients and offer the opportunity to monitor the progression of tau pathology along with muscle impairment.

In conclusion, [^18^F] THK5317-PET can reveal muscular tau deposition in vivo, which provides a new and noninvasive diagnostic method for sIBM and offers the opportunity to monitor the progression of tau pathology along with muscle impairment. While due to only one patient included, our findings must be considered as preliminary and further study in a larger number of participant patients is needed to confirm these findings.

## Acknowledgments

We thank the patient and her family for their kind cooperation.

## Author contributions

**Conceptualization:** Ke Li, Qiang Shi.

**Data curation:** Yutong Zhang, Ke Li.

**Formal analysis:** Yutong Zhang.

**Funding acquisition:** Ke Li, Chuanqiang Pu, Qiang Shi.

**Investigation:** Yutong Zhang.

**Methodology:** Haodan Dang, Jiajin Liu.

**Project administration:** Qiang Shi.

**Resources:** Ke Li.

**Supervision:** Chuanqiang Pu.

**Visualization:** Haodan Dang, Jiajin Liu.

**Writing – original draft:** Yutong Zhang, Ke Li.

**Writing – review & editing:** Qiang Shi.
